# Tackling SARS-CoV-2: proposed targets and repurposed drugs

**DOI:** 10.4155/fmc-2020-0147

**Published:** 2020-06-22

**Authors:** Siddhi Joshi, Maithili Joshi, Mariam S Degani

**Affiliations:** ^1^Department of Pharmaceutical Sciences & Technology, Institute of Chemical Technology, Nathalal Parekh Marg, Mumbai, 400019, Maharashtra, India

**Keywords:** antivirals, COVID-19, drug targets, repurposed drugs, SARS-CoV-2, therapeutics

## Abstract

The SARS-CoV-2 pandemic, declared as a global health emergency by the WHO in February 2020, has currently infected more than 6 million people with fatalities near 371,000 and increasing exponentially, in absence of vaccines and drugs. The pathogenesis of SARS-CoV-2 is still being elucidated. Identifying potential targets and repurposing drugs as therapeutic options is the need of the hour. In this review, we focus on potential druggable targets and suitable therapeutics, currently being explored in clinical trials, to treat SARS-CoV-2 infection. A brief understanding of the complex interactions of both viral as well as host targets, and the possible repurposed drug candidates are described with an emphasis on understanding the mechanisms at the molecular level.

## Graphical abstract

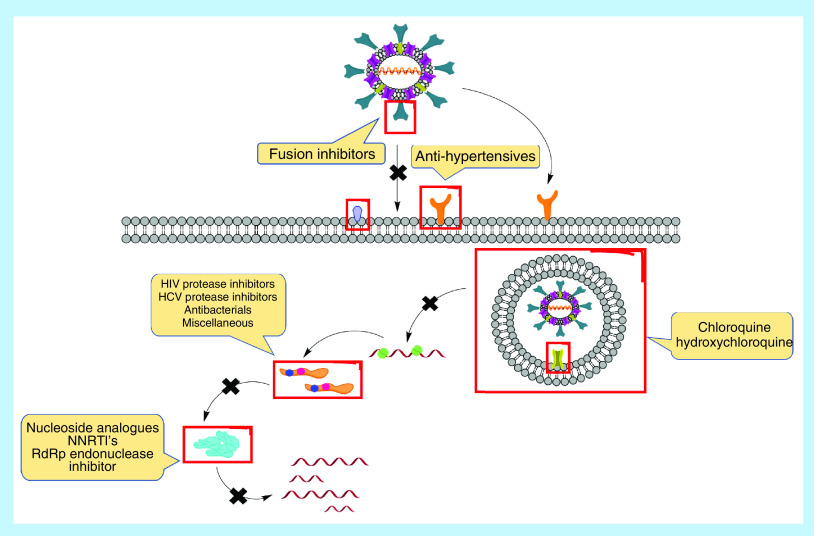

The coronavirus disease 2019 (COVID-19) is a global pandemic caused by the novel severe acute respiratory syndrome coronavirus 2 (SARS-CoV-2) that has posed serious threats to humans, and adversely affected the health and economy of many countries. The SARS-CoV-2 infection emerged in December 2019. Soon, it spread across the globe causing a pandemic and has become a major health concern [1,2]. As of 1^st^ June 2020, the WHO reported a total of 6,057,853 cases worldwide with 371,166 deaths due to COVID-19 [3].

Coronaviruses belong to the *Coronoviridae* family, consisting of four genera, namely *Alphacoronavirus, Betacoronavirus, Gammacoronavirus and Deltacoronavirus*. The SARS-CoV-2 belongs to the *Betacoronavirus* genera [4]. Like all coronaviruses, SARS-CoV-2 is a positive stranded, non-segmented RNA virus. Coronaviruses have the largest genome among all of RNA viruses, ranging from 27–32 kD [5].

Clinical features of SARS-CoV-2 infection are similar to SARS and MERS. Common symptoms of SARS-CoV-2 infection include fever, fatigue, dry cough and dyspnea, which may progress into acute respiratory distress syndrome (ARDS) causing death [6]. SARS-CoV-2 is highly contagious and has exhibited transmission through fomites, cough and cold droplets and human contact [2]. Some of the prevention strategies include washing hands regularly with soap and water or alcohol-based hand rubs, covering nose and mouth while coughing and sneezing and avoiding close contact with people who are unwell. Self-isolation at home has been advised if a person is sick [7]. Currently, vaccines to prevent COVID-19 are under development, while chemical entities like chloroquine, remdesivir, lopinavir and ritonavir have shown promising results in cell studies and clinical trials (ClinicalTrials.gov: NCT04283461]) [8,9].

This review focuses on repurposed small molecules as a therapeutic intervention for COVID-19 along with an understanding of the main targets; both viral and host based.

## Structure & replication cycle of SARS-CoV-2

### Structural features

Coronaviruses, including SARS-CoV-2 are spherical and have diameters ranging from 65 to 125 nm [10]. The SARS-CoV-2 has a spherical and pleomorphic form [11].

SARS-CoV-2 has a single-stranded, nonsegmented, positive sense RNA. Two thirds of the genomic material constitute the replicase gene, which codes for 16 nonstructural proteins (nSPs) important for viral RNA replication and transcription. The rest of the genome codes for structural proteins (SPs) of the virus [2,12].

**Figure 1.**
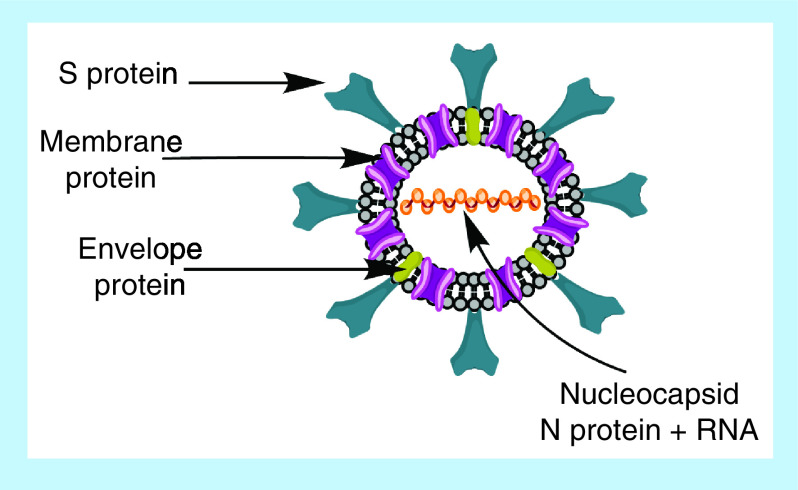
Various proteins associated with SARS-CoV-2.

Like all coronaviruses, the major structural proteins of SARS-CoV-2 are spike glycoprotein (S), nucleocapsid proteins (N), membrane proteins (M) and envelope proteins (E) [6,12–15]. The spikes are seen as protrusions from the virus surface, thus giving the appearance of a crown to the virus, as seen in Figure 1. Hence the name ‘coronavirus’. The S proteins are homotrimeric glycoproteins, responsible for attachment and penetration of the virus into the host cell. They are also the major inducers of immune responses [6]. The S protein is made up of 2 subunits – S1 and S2. S1 is responsible for binding to the host cell receptor and S2 is involved in fusion of the viral and cell membranes [16]. The S protein has to be primed for activation and entry of the SARS-CoV-2 into the host cell [17–20].

The RNA genome is packaged inside the helical capsid formed by the N proteins, which is further surrounded by a phospholipid bilayer envelope [6,12]. The M and E proteins are embedded in the viral envelope and are involved in virus assembly post RNA translation. M proteins interact with other structural proteins, aid in envelope formation and bud release [2,6,12]. The E proteins function as ion channels and are also involved in the assembly of the virus during replication [12].

### Replication cycle of SARS-CoV-2

**Figure 2.**
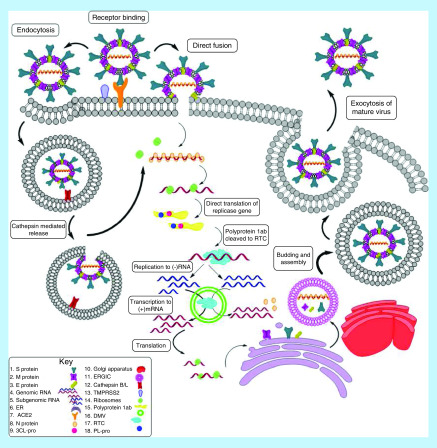
Replication cycle of SARS-CoV-2. DMV: Double membrane vesicle; ER: Endoplasmic reticulum; RTC: Replicase–transcriptase complex.

As depicted in Figure 2, the replication cycle of SARS-CoV-2 can be broadly divided into three processes – viral entry, viral RNA replication and lastly, viral assembly and exit from the host cell.

Several host cell proteins have been suggested to associate with the viral S protein of SARS-CoV-2, facilitating viral invasion into cells. Early on, it was widely reported that SARS-CoV-2 uses angiotensin-converting enzyme 2 (ACE2) as a receptor for entry into the host cell, similar to SARS-CoV [16,18,20,21]. However more recent studies indicate two other receptors, GRP78 and CD147 may also be involved. CD147, a transmembrane glycoprotein on the surface of the host cell has been proposed as an alternative pathway for infection [22–24]. Also, an *in silico* study predicted the binding of S protein to GRP78; a chaperone heat shock protein in cells [25].

The receptor binding domain (RBD) of S1 subunit binds to ACE2 on the cell surface. The inactive S needs to be cleaved at the S1/S2 and S2' sites for further viral entry into the host cell. Studies on other coronavirus S proteins have reported many host proteases involved in the spike activation like TMPRSS2, cathepsin L, B, furin and trypsin [14,19,20]. Depending on their availability, the mechanism of viral entry differs. Previous reports have recorded entry of SARS-CoV either via an endocytic pathway or direct fusion with plasma membrane. Surface proteases of the host like TMPRSS2 or exogenous trypsin can cleave the S protein, triggering fusion of viral and plasma membranes, facilitating direct release of the viral genome into the cytoplasm. SARS-CoV can also enter the cell by endocytosis post receptor binding. Endosomal cathepsin B and L cleave the S protein, resulting in fusion of the viral and endosomal membranes and subsequent release of viral genome [14,18–20,26,27].

The mechanism of entry for SARS-CoV-2 has not been well elucidated yet. However, an *in vitro* study by Hoffmann *et al.* reported that SARS-CoV-2 entry involved S activation by TMPRSS2 [17,28]. Also, SARS-CoV-2 can use the endocytic pathway to infect cells [17]. Similar results were reported by Xiuyuan Ou *et al.* whose *in vitro* study showed that SARS-CoV-2 mainly entered host cells through endocytosis and that cathepsin L played a critical role in activating S protein [18]. Apart from TMPRSS2 and cathepsin L, it has also been reported that the S1/S2 cleavage site of SARS-CoV-2 could be recognised by furin, a feature missing in SARS-CoV [5,29]. Also, exogenous trypsin could increase the infectivity of SARS-CoV-2 *in vitro* by cell–cell fusion [17,18,29]. All these proteases could play a role in activating S protein, facilitating viral access to the cytoplasm.

Once the virus enters the host cell, it releases the RNA genome into the cytoplasm. The replicase gene on the positive sense RNA is directly translated to a polyprotein, pp1ab. This polyprotein pp1ab is then cleaved by viral proteases including 3-chymotrypsin like protease (3-CL pro) and papain like protease (PL-pro) to form 16 individual nSPs [30]. nSPs, on assembly, form the RNA replicase-transcriptase complex (RTC). The RTC localizes near the ER in a double membrane vesicle (DMV) [14]. The RTC is involved in replicating and transcribing the RNA genome into a set of negative sense RNAs. Full-length negative sense RNAs act as templates for transcribing the genomic RNA, ready to be incorporated into the virion. However, discontinuous replication by RTC forms partial, negative sense, subgenomic RNA strands. These act as templates for transcription of subgenomic mRNA which code for the structural proteins (SPs) of the virus [2,12,14,31–33].

The mRNA is translated using the cellular ribosomes to form the M, N, E, S proteins. The SPs are inserted into the ER, from where they are transported to the ER-Golgi Intermediate Complex (ERGIC). The N protein enclosing the genomic RNA is assembled with other SPs in ERGIC, forming viral buds. The mature virions are transported to the cell membrane in a vesicle and they exit the host by exocytosis [2,12,14,31,32].

## Targets & therapeutics against SARS-CoV-2

**Figure 3.**
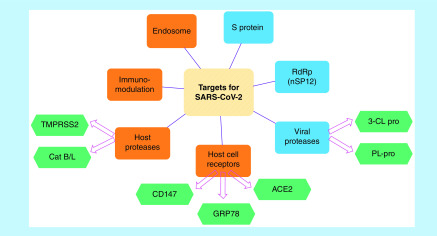
Potential druggable of targets for treatment of COVID-19. S: Spike.

Currently, there is no US FDA approved drug for COVID-19 [34]. Presently, therapy focuses on offering symptomatic relief combined with the use of existing anti-viral agents and host response modulators [2,35]. Anti-viral agents used for HIV, SARS, HCV, influenza and malaria are being repurposed for SARS-CoV-2 infection. Drugs like remdesivir, chloroquine, ribavirin, lopinavir and ritonavir have shown some efficacy in treating COVID-19 and are under clinical studies [27,36,37]. Several other drugs and potential druggable targets are being explored for their efficacy against SARS-CoV-2 [33]. Presently, most of the drugs under clinical trials show effect against SARS-CoV-2 by targeting viral replication or inhibiting viral proteases. Successful druggable targets include RNA dependent RNA polymerase (RdRp), viral proteases like 3-CL pro, PL-pro and endosomal pathway inhibitors as summarized in Figure 3 [33,38,39]. Insights into detailed mechanism of viral replication and the viral structure can help in identifying further potential targets for therapeutics.

### Potential targets against SARS-CoV-2 entry into the host cell

#### Blocking of spike protein

As described before, the spike (S) protein plays a vital role in viral entry into the host cell. Therefore, it is an attractive target for blocking SARS-CoV-2 infection.

**Figure 4.**
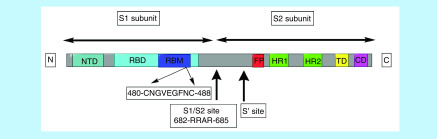
The subunits of S protein-1 unit forms the N terminal region of the S protein. It consists of an NTD and a RBD. RBM in the domain binds to the receptor on the host cell membrane. S2 subunit is the C terminal region consisting of a FP, HR1 and HR2, TD and CD. Residues of Region IV (Cys480-Cys488) in the RBD of the S protein include 9 residues- Cys480, Asn481, Gly482, Val483, Glu484, Gly485, Phe486, Asn487, Cys488 that bind to GRP78 of the host. CD: Cytoplasmic domain; FP: Fusion peptide; HR1: Heptad Repeats 1; HR2: Heptad Repeats 2; NTD: N terminal domain; RBD: Receptor binding domain; RBM: Receptor binding motif; TD: Transmembrane motif.

So far, the S protein is the most widely characterized protein of SARS-CoV-2 as depicted in Figure 4 [5,18,21].

**Figure 5.**
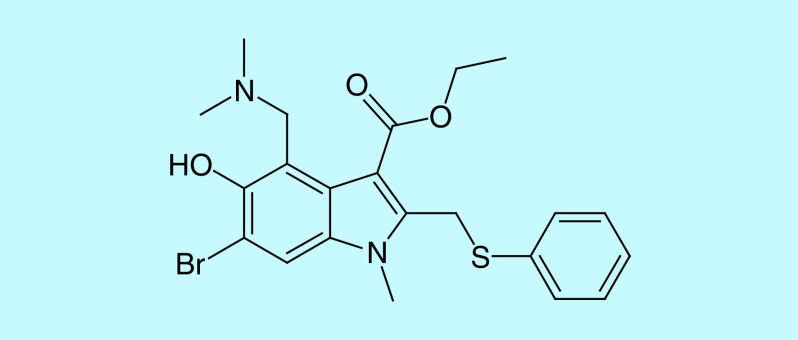
Umifenovir chemical structure.

**Figure 6.**
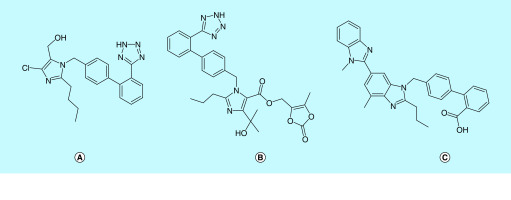
ATR1 blockers. **(A)** Losartan, **(B)** olmesartan and **(C)** telmisartan.

The S protein is made up of two subunits, S1 and S2. The coronavirus first binds to the receptor on the host cell surface through the RBD of S1 subunit, and then fuses the viral and host membranes through the S2 subunit. The S protein has to be activated to facilitate viral entry. It is cleaved by host cell proteases at two sites; S1/S2 priming site at the junction of the two subunits and S2' site- the activating cleavage site located upstream of FP [17,18,29,40]. Once the sites are cleaved, the exposed fusion peptide inserts itself into the plasma membrane. The HR1 and HR2 domains interact to form the 6 helical bundle (6HB) fusion core, ultimately leading to fusion of viral-plasma membranes [29,40]. ACE2 on the cell membrane of the host cell, has been reported as the receptor for binding of S1 subunit for all SARS-CoV [5,18,20,21]. The SARS-CoV-2 spike is likely to bind 10- to 20-times more than SARS-CoV to ACE2 [15,16,40]. This might account for the high infectivity of SARS-CoV-2. The receptor binding domain (RBD) is therefore an attractive target for developing neutralizing antibodies, viral inhibitors and vaccines [5].

A study in March 2020 by Tai W. *et al.*, reported a recombinant RBD protein which competitively binds to ACE2 receptors, thus inhibiting infection of SARS-CoV-2 in cells [41]. Another molecular modelling study by Ibrahim *et al.* also revealed that the spike protein of SARS-CoV-2 binds to the GRP78 substrate binding domain β (SBDβ) during conditions of cellular stress. Four regions of the S protein were docked and Region IV was suggested to be the recognition site for the cell surface GRP78 SBDβ [25]. Region IV that lies in the receptor binding motif (RBM) of SARS-CoV-2 is 9 amino acid long sequence- 480Cys-Asn-Gly-Val-Glu-Gly-Phe-Asn-Cys-488 of which Cys480, Val483, Phe486 and Cys488 are active residues, critical for binding [25]. This region could also be targeted to inhibit the invasion of SARS-CoV-2 into the host cells via alternative pathways.

Since the RBD plays a crucial role in recognising and binding to the receptors on the host cell, it has emerged as a major target for the development of both therapeutics and vaccines [42]. However, the amino acid sequence of RBD is susceptible to changes through missense point mutations which may alter the stability and interactions of RBD with the receptors. Some studies have identified these missense mutations in different isolates of COVID-19 that have either improved or reduced the binding affinity of RBD and ACE2 [43–46]. Hence, mutations seen in the RBD of the spike protein may have a significant impact on the process of vaccine development.

Another recent study has also targeted- blocking the 6HB core of the HR1 and HR2 domains in the S2 subunit which results in fusion of plasma and viral membranes. EK1C4, a lipopeptide, with a cholesterol moiety was the most potent fusion inhibitor against SARS-CoV-2 S protein-mediated membrane fusion. It was shown to inhibit the formation of the 6HB fusion core. Thus, it could be a potent inhibitor of S protein mediated membrane fusion in SARS-CoV-2 [40].

Additionally, umifenovir also known as arbidol has been used to treat influenza and had previously shown efficacy against SARS and MERS. It has been employed in clinical studies for SARS-CoV-2 in China (ClinicalTrial.gov: NCT04252885). Although the mechanism of its anti-viral action is not well elucidated in SARS-CoV-2, it has been proposed as a fusion inhibitor of viral and host cell membranes in influenza [33,37,47,48].

#### Targeting host cell receptors

##### ACE2 & antihypertensives

The binding of SARS-CoV-2 S1 subunit to ACE2 is the point of entry into the host [21]. ACE2 and its closely related homologue ACE are involved in vasodilation and vasoconstriction, respectively. ACE catalyses the conversion of angiotensin I (AT1) to angiotensin II (AT2)-a vasoconstrictor, the peptide which binds to and activates ATR1 (angiotensin receptor I) to constrict blood vessels, thereby elevating blood pressure. Conversely, ACE2 catalyses the conversion of AT2 to angiotensin 1-7 a vasodilator. ACE2 is widely expressed in the alveolar epithelial cells of the respiratory tract [49–51]. It has been demonstrated that binding of coronavirus spike proteins downregulate ACE2 expression in cells. Hence, less ACE2 is capable of converting angiotensin II to the vasodilator heptapeptide angiotensin 1-7. This could contribute to more hypertension and severe lung injury. Anti-hypertensives block AT1 mediated vasoconstriction and increase ACE2 expression. This may have a protective effect on the lungs rather than putting them at a higher risk of developing SARS-CoV-2 [49,50,52]. Therefore, the role of antihypertensives like losartan, olmesartan and telmisartan could be further evaluated against SARS-CoV-2 infection.

Chloroquine and hydroxychloroquine have been proposed to alter post translational modifications of ACE2 receptors in the host, thereby blocking SARS-CoV-2 infection. Chloroquine and hydroxychloroquine could inhibit the glycosylation of ACE2 receptors, changing its structure and therefore blocking the binding of S protein to it [2,32,39].

##### Inhibiting CD147 receptor

CD147, also known as basigin (BSG) or extracellular matrix metalloproteinase inducer (EMMPRIN) is a transmembrane glycoprotein belonging to the immunoglobulin superfamily. It is expressed in several types of cells in the lungs like epithelial cells, macrophages and type II pneumocytes at the edges of fibrotic zones. CD147 functions as an upstream stimulator of matrix metalloproteinases (MMP's) [23]. Previously, CD147 has been associated with HIV-1, SARS-CoV, plasmodium infections and development of tumors [24]. A recent *in vitro* study by Wang *et al.* showed that SARS-CoV-2 infected cells via binding of S protein and CD147 [22].

A humanized antibody-mepolizumab has been shown to reduce the infection of SARS-CoV-2 significantly *in vitro*, and hence has been employed in clinical trials (ClinicalTrials.gov: NCT04275245) [22,53]. The anti-bacterial macrolide; azithromycin has demonstrated reduced levels of MMP expression and activity in other studies. This may be related to a reduced expression of CD147 in cells [23].

##### Inhibiting GRP78 receptor

GRP78, also known as immunoglobulin heavy chain BiP is a member of the HSP70 family. Under normal physiological conditions, it is found in the lumen of the ER where it functions as a molecular chaperone and is involved in the degradation of misfolded proteins [54]. However, its overexpression, initiated during cell stress, leads to the translocation of GRP78 to the plasma membrane. At the cell surface, it can interact with several ligands including RBD of the viral S protein, acting as a multifunctional receptor for viral entry [25,54]. Previously, GPR78 has been reported to mediate viral entry and development of the envelope proteins in the Ebola virus, Zika virus, influenza virus, HCV and MERS-CoV [54].

An *in silico* study by Ibrahim *et al.* reported the interaction of the S protein of SARS-CoV-2 and surface GRP78 [25]. Thus, similar to MERS-CoV, the SARS-CoV-2 S protein can recognize and bind to the GRP78 SBDβ, facilitating viral entry when the cell is under stressful conditions. Natural products like daidzein, genistein, formononetin, biochanin A, estriol, estradiol and hydroxycortisone have shown to block GRP78 SBDβ in a molecular docking study [55]. Thus, molecules targeting GRP78 could be explored for inhibiting SARS-CoV-2 infection.

#### Inhibiting host cell proteases

The inactive S protein is cleaved by host cell proteases like TMPRSS2, cathepsin B, L, furin, and trypsin-like proteases to trigger fusion of the cell and viral membranes [19,20,26]. TMPRSS2 is a serine protease on the surface of the cell membrane. Previous studies on SARS-CoV had shown that TMPRSS2 played an important role in its entry into the host cell [14,19,20]. Similarly, the entry of SARS-CoV-2 in the host cell depends upon the cleaving activity of TMPRSS2 [17,28]. SARS-CoV-2 exhibited increased infectivity in cells in the presence of TMPRSS2 [18]. Also, TMPRSS2 inhibitors like camostat mesylate have shown to inhibit SARS-CoV-2 infection *in vitro* [17,18,26,28]. Interestingly, an *in silico* study also demonstrated higher binding affinity and lower binding energy of remdesivir to TMPRSS2 which could help block its activity [33].

The spike glycoprotein has a potential cleavage site for furin proteases. Furin is a Type I transmembrane proteolytic enzyme present in host cells. It is a peptidase which essentially converts inactive precursor proteins into their active counterparts [56]. SARS-CoV-2 has a 4 amino acid linkage [682-Arg-Arg-Ala-Arg-685] at the junction of S1 and S2 subunit which is missing in SARS-CoV. This linkage can be recognized as a substrate by furin for proteolysis [18,29]. Furin is well expressed in the cells of the respiratory tract, hence increasing the infectivity of SARS-CoV-2. Thus, furin inhibitors could be explored for their efficacy against SARS-CoV-2 [29,57]. The linkage makes the S1/S2 site polybasic, which indicates high cleavability [16,20]. Therefore, it would be efficiently cleaved by trypsin as well. The linkage makes the S protein much more susceptible to cleavage than that of SARS-CoV. This might account for its high transmissibility.

Additionally, SARS-CoV-2 can enter the host via endocytosis. Cathepsins B and L belong to the papain subfamily of cysteine proteases. Both cathepsin B and L are located predominantly in the endolysosomal vesicles and cathepsins have been shown to be critical for SARS-CoV entry through endocytosis [58]. Cathepsin L-a cysteine protease in the endosome is responsible for cleaving the S proteins and subsequent fusion of endosomal-viral membranes [14,18]. Hoffmann *et al.* reported that only blocking TMPRSS2 could not inhibit SARS-CoV-2 infection fully, implying cathepsin-dependent activity of the virus [17,18]. Cathepsin B and L inhibitors like E-64D, K11777 (a vinyl sulfone cysteine protease inhibitor), SID26681509 were successful in blocking SARS-CoV-2 infection *in vitro* [17,18,26].

#### Impairing the endosomal pathway of viral entry

The endosomes need a lower pH for their maturation and efficient functioning. Compounds elevating the endosomal pH, inhibit the fusion of endosomal-viral membrane and block the release of the viral genome [59]. Major drug candidates-chloroquine and its derivatives have been proposed to show efficacy against the infection via this mechanism. Chloroquine and hydroxychloroquine are weak, diprotic bases. They increase the endosomal pH, inhibiting the activity of cathepsins and blocking membrane fusion. Chloroquine may also possibly block the maturation of endosomes, inhibiting the transport and release of SARS-CoV-2 genome [2,32,39,60–62]. Thus, the trafficking and release of the viral genome through endosomes is blocked.

Hydroxychloroquine, a less toxic derivative of chloroquine has also shown good therapeutic potential against SARS-CoV-2 [60,63,64]. Also, chloroquine and hydroxychloroquine can modulate immune responses. Hydroxychloroquine is used to treat autoimmune diseases like rheumatoid arthritis as an anti-inflammatory agent. SARS-CoV-2 patients have shown increased cytokine and inflammatory responses. Hence this property could be beneficial [60,63].

**Figure 7.**
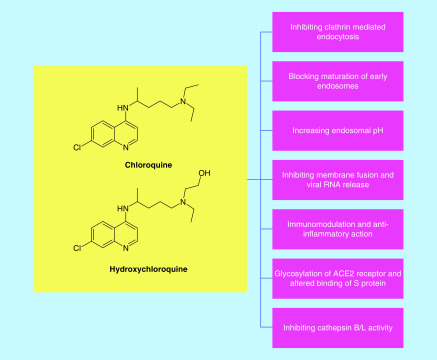
Summary of proposed mechanisms of chloroquine and hydroxychloroquine.

The proposed mechanisms by which chloroquine and hydroxychloroquine exhibit therapeutic benefit has been summarised in Figure 7.

### Potential targets against viral replication

The replication of SARS-CoV and SARS-CoV-2 is mediated by the replicase polyprotein. The replicase pp1ab of SARS-CoV-2 is processed by two viral proteases, PL-pro and 3-CL pro, to generate 16 replicase products, called nonstructural proteins (nSPs) [65]. These 16 nSPs then assemble to form replicase–transcriptase complex (RTC) which is involved in RNA replication and transcription. One of the nSPs, nSP12 plays a crucial role in the RNA replication of SARS- CoV-2 as described in the following section.

#### Inhibition of RdRp

**Figure 8.**
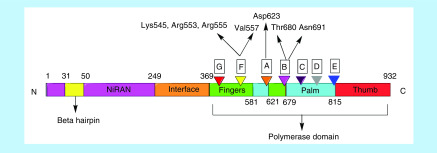
nSP12 is a 932 amino acid long protein. The domains identified so far are a β hairpin at the N-terminal followed by a nidovirus-unique N-terminal extension domain which functions as a nidovirus RdRp-associated nucleotidyltransferase. An interface domain connects the NiRAN domain to the polymerase domain. The polymerase domain is further divided into three subdomains including a fingers subdomain, a palm subdomain and a thumb subdomain. The polymerase domain is composed of seven conserved motifs: A, B, C, D, E, F, G which contribute to the activity of the polymerase.

One of the most important nSPs formed as a result of cleaving pp1ab is nSP12. nSP12 functions as RdRp [12,33,36]. RdRp is involved in directly replicating the positive sense viral RNA to a set of negative sense genomic and subgenomic RNA strands. These negative templates are again transcribed by RdRp to form subgenomic mRNAs and genomic viral RNA [66,67]. For RNA viruses, RdRp presents an optimal target due to its crucial role in RNA synthesis, lack of host homologs and high sequence and structural conservation. RdRp of SARS-CoV and SARS-CoV-2 share a similarity of 98%. RdRp needs nSP7 and nSP8 as cofactors for its enhanced activity. Though other viral nSPs are also necessary to carry out replication and transcription, the nSP12-nSP7-nSP8 complex is the minimal complex required for polymerization of nucleotides [68–70]. As depicted in Figure 8, the polymerase domain comprises of seven motifs which are involved in the binding of the template strand and nucleotides and catalyzing polymerization [69]. The nucleoside triphosphate (NTP) entry channel is formed by a set of hydrophilic residues that includes Lys545, Arg553 and Arg555 in motif F [68,69]. Residues Thr680 and Asn691 of motif B are likely to form hydrogen bonds with the 2′ hydroxyl group of the incoming NTP while Asp623 in motif A is involved in forming a hydrogen bond with the 3′ -OH group of the incoming NTP. The RNA template enters the active site through a groove clamped by motifs F and G. Motif E and the thumb subdomain support the primer strand [68,69]. Val557 of motif F appears to be a crucial residue of the active site involved in base pairing of the NTPs [68–70].

**Figure 9.**
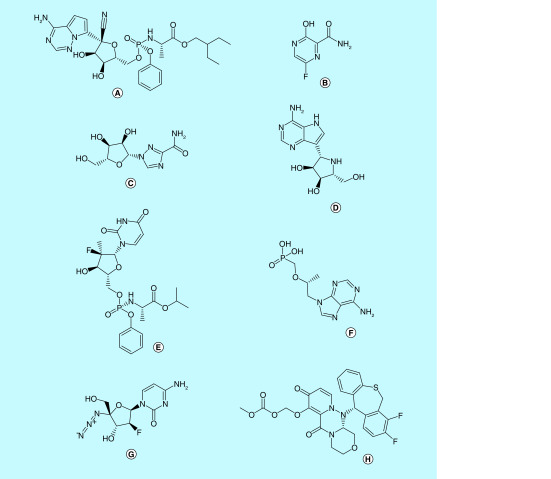
RdRp inhibitors. **(A)** Remdesivir, **(B)** favipiravir, **(C)** ribavirin, **(D)** galidesivir, **(E)** sofosbuvir, **(F)** tenofovir, **(G)** azvudine and **(H)** baloxavir marboxil.

RdRp has been a well explored target for several RNA viruses including SARS, Ebola and MERS [71,72]. Nucleotide analogs (NA) like remdesivir, favipiravir, galidesivir and ribavirin have been reported to show efficacy against SARS-CoV-2 by blocking RdRp activity. RdRp recognises these nucleotide analogs as substrates, leading to the termination of the RNA replication. NAs incorporated into the viral RNA exhibit their anti-viral activity through the following mechanisms of action (MoA). First, the nucleoside analog lacks the 3′-OH required for chain extension or the incorporation of the analog causes a disruption in the nascent RNA structure, stopping its further synthesis. Second, the incorporation of several NTPs throughout the nascent RNA causes an increase in mutations. This ultimately leads to the formation of a nonviable genome through a process called lethal mutagenesis [73,74].

Remdesivir, a 1′ cyano substituted adenosine analog, has shown an excellent profile against SARS-CoV-2. It has been used to successfully treat the first COVID-19 patient in the USA and is currently under clinical trials (ClinicalTrials.gov: NCT04292899, NCT04292730 and NCT04280705) [2,36,37,72]. Remdesivir is a phosphoramidate prodrug which on hydrolysis forms the active adenosine analog [37]. Remdesivir competes with its natural counterpart ATP, to be paired opposite UTP on the template. Remdesivir does not terminate the chain immediately after its incorporation, however if remdesivir triphosphate is incorporated at a position i, then it results in delayed chain-termination between position i+3 and i+5 [73,75].

Favipiravir or T-705 is a guanine analog which has been under clinical trials for Ebola virus and influenza. Favipiravir is a prodrug which is converted to its active triphosphate form by intracellular phosphoribosylation [76,77]. It has shown to inhibit SARS-CoV-2 infection in cells and is being tested in clinical trials [37,72]. Interestingly, a clinical study in China reported favipiravir to exhibit greater activity against COVID-19 than the widely approved lopinavir/ritonavir combination for SARS-CoV-2 [72,78]. However, another clinical study analysed the efficacy of umefenovir against favipiravir among patients with moderate COVID-19. It reported no significant difference in the recovery rate of Day 7 among patients. However, favipiravir did show improved latency to relief for pyrexia and cough [79].

Ribavirin is a broad-spectrum antiviral drug used against HCV, RSV, SARS and MERS. It is a guanosine derivative which is currently being tested in clinical trials with lopinavir/ritonavir and interferons [9,36,37,48]. Ribavirin is also a prodrug which undergoes intracellular phosphorylation to become active [76,80].

Galidesivir, another prodrug of an adenosine analog, originally developed for HCV has shown effect against several RNA viruses including SARS and MERS [9,37,48]. Tenofovir, an adenosine analog, is a potent HIV1 and HCV polymerase inhibitor has shown to bind tightly with SARS-CoV-2 RdRp in molecular docking studies [38,81]. The combination of tenofovir and emtricitabine used for the treatment of HIV, is currently under clinical trials for SARS-CoV-2 [9,48]. Sofosbuvir, a uridine analog which inhibits HCV RdRp, has shown similar promising results in a docking study for SARS-CoV-2 [82,83].

However, nucleotide analogs can be excised during RNA replication by the exonucleases (ExoN). The N terminal domain of nSP14 functions as the exonuclease for SARS-CoV-2 and is involved in proofreading activity [12]. Analogs like ribavirin are readily excised from the RNA by ExoN, thereby rendering it less effective for SARS-CoV infection [73]. However, an *in silico* model studying the interaction of nSP14 of SARS-CoV and remdesivir triphosphate reported that it may be less efficiently excised by ExoN, making remdesivir more efficacious [73].

Another anti-viral drug used for the treatment of influenza-Baloxavir marboxil is under a randomized, controlled clinical trial in China for SARS-CoV-2 (ChiCTR2000029544 and ChiCTR2000029548). Baloxavir marboxil is a prodrug which is hydrolyzed to form the active drug-baloxavir acid. Baloxavir acid is a potent inhibitor of the RdRp acidic endonuclease domain of the influenza virus. Thus, it inhibits the process of viral replication [84].

Azvudine-a nucleoside reverse transcriptase inhibitor (NRTI) exhibiting antiviral activity against HIV, HCV and HBV has also been employed in SARS-CoV-2 clinical trials in China (ChiCTR2000030487, ChiCTR2000029853 and ChiCTR2000030424). Azvudine is a cystidine analog which undergoes phosphorylation to form active NRTI triphosphate (NRTI-TP). NRTI-TP is then incorporated into the primer strand by reverse transcriptase (RT), thus inhibiting viral DNA synthesis [85].

#### Inhibiting viral proteases

##### 3-CL pro

3-CL pro is responsible for processing the replicase polyproteins at 11 sites, thus giving rise to nSP4 to nSP16. It is also called M pro. 3-CL pro also mediates the maturation of the non-structural proteins, which is an essential step in the replication of the virus [4,33].

**Figure 10.**
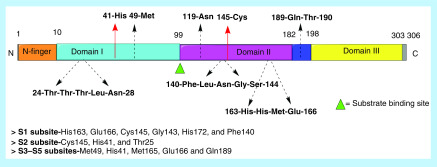
The linear structure of a 3-CL pro monomer consists of 3 domains--Domain I, Domain II and Domain III [89]. The N-terminal domain comprises of Domain I and II. The N-terminal residues 1-9 form the N-Finger. It is involved in the dimerization and formation of the active site of 3-CL pro. Domain I (10–99 residues) and Domain II (100–182) form an antiparallel β-barrel structure with 13 β strands. The active site of the enzyme is located in a cleft between the domains I and II. SARS-CoV-2 3-CL pro also contains the same catalytic dyad as SARS-CoV 3-CL pro – His41 and Cys145. A long loop of residues 185–191 link Domain II and III. Domain III (198–303) makes up the C-terminal domain of 3-CL pro. Domain III consists of a globular cluster of 5 α-helices. It is involved in regulating dimerization of the enzyme [86,90,91].

As depicted in Figure 10, the monomer of SARS-CoV-2 3-CL pro is a 306 amino acid long protease. It has shown a 96% sequence similarity with 3-CL pro of SARS-CoV [86]. Only 12 out of the 306 residues are different from those on SARS-CoV. None of these 12 variant residues are a part of the binding pocket, hence they may not be involved in any major role [87]. Also, all the 11 3-CL pro cleavage sites on polyprotein pp1ab are highly conserved [88]. A typical substrate sequence for 3-CL pro is Leu-Gln ↓ (Ser, Ala, Gly) where it cleaves the polyprotein at ↓ [86,88]. Since there are no known human proteases with a similar cleaving specificity, 3-CL pro inhibitors are less likely to be toxic [86].

**Figure 11.**
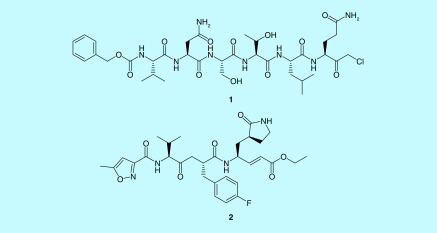
Compound 1 and 2. Compound 1: a peptide inhibitor Cbz-Val- 35 Asn-Ser-Thr-Leu-Gln-CMK and compound 2: rupintrivir.

**Figure 12.**
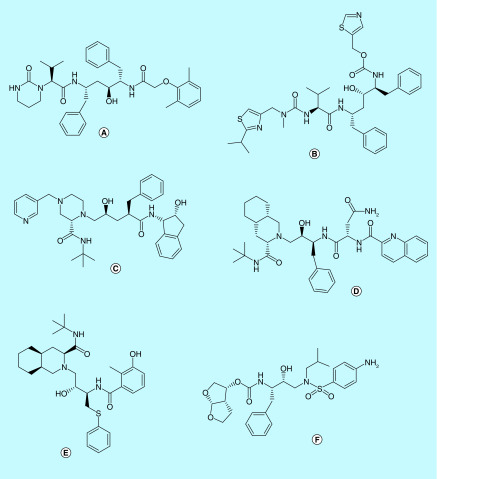
HIV protease inhibitors. **(A)** Lopinavir, **(B)** ritonavir, **(C)** indinavir, **(D)** saquinavir, **(E)** nelfinavir and **(F)** darunavir.

**Figure 13.**
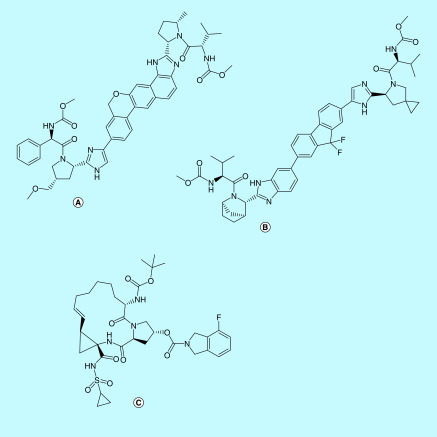
HCV protease inhibitors repurposed for SARS-CoV-2. **(A)** Velpatasvir, **(B)** ledipasvir and **(C)** danoprevir.

Dimerization is necessary for the catalytic activity of 3-CL pro [86]. The active site of 3-CL pro contains the catalytic dyad – His41 and Cys145. This catalytic dyad is conserved in 3-CL pro of SARS-CoV and SARS-CoV-2 [86,90,92]. Apart from the catalytic site, there are two deep seated sites-S1 and S2 and 3 shallow site-S3, S4, S5. The deep sites are involved in electrostatic and hydrophobic interactions. Important residues in these binding sites include Phe140, Leu141, Asn142, Gly143, Ser144, Met165, Glu166, Gln189 and Thr190 [93]. Residues like Thr24 to Asn28 and Asn119 are involved in binding to inhibitors by forming hydrogen bonds [92].

The first x-ray structure of SARS-CoV 3-CL pro with a peptide inhibitor chloromethylketone (CMK; Cbz-Val- 35 Asn-Ser-Thr-Leu-Gln-CMK: compound 1) was elucidated in 2003 [94]. Compound 2, a peptidomimetic antiviral- rupintrivir, showed similar binding affinity to 3-CL pro of rhinovirus, which is very similar to the 3-CL pro of coronavirus. Rupintrivir is an experimental molecule developed for common cold. Therefore, peptide inhibitor CMK and rupintrivir have been used as starting points to develop more efficacious 3-CL pro inhibitors [94,95].

Some of the peptidomimetics and small molecules mainly used in the inhibition of 3-CL pro are discussed below:

**HIV protease inhibitors**
  Lopinavir and ritonavir, originally HIV protease inhibitors, are being widely used in combination for treating SARS-CoV-2 infection. These have been proposed to inhibit 3-CL pro in SARS and SARS-CoV-2, thereby inhibiting the viral replication [37,48,72]. Docking studies have shown that lopinavir and ritonavir have a 3-CL pro binding affinity comparable to that of HIV proteases [4,89,96]. Thr24 to Asn28 and Asn119 formed key residues of the binding pocket for lopinavir and ritonavir [92].
  Lopinavir/ritonavir have also been employed in several clinical trials in China and South Korea and have shown some promise in some of the trials (ClinicalTrials.gov: NCT04276688 and NCT04307693) [48,72,97]. It has been proposed that since HIV proteases are aspartate proteases, whereas 3-CL pro and PL-pro are cysteine proteases, the efficacy of lopinavir/ritonavir has been debatable [37]. Nevertheless, till a better drug candidate is found, lopinavir/ritonavir treatment remains a potentially efficacious combination. Other HIV protease inhibitors like darunavir, saquinavir, indinavir, nelfinavir have shown good binding affinities to 3-CL pro in *in silico* studies. It had been reported that darunavir had exhibited efficacy against SARS-CoV-2 *in vitro* as well [4,72,98]. The efficacy of darunavir for COVID-19 is being tested in a Phase III clinical trial (ClinicalTrials.gov: NCT04252274). However, a report from Jansenn Pharmaceutica (Johnson & Johnson) stated that darunavir did not exhibit efficacy against SARS-CoV-2 *in vitro* at clinically relevant concentrations and that the combinatory therapy of darunavir and cobicistat was ineffective in clinical trials for SARS-CoV-2 [99]. Hence, there is a lack of evidence for the use of darunavir against SARS-CoV-2.

**HCV protease inhibitors**
  Danoprevir is a potent HCV protease inhibitor. It has been tested in clinical trials in combination with ritonavir to suppress viral replication in patients with moderate SARS-CoV-2 infection (ClinicalTrials.gov: NCT04291729) [100]. Velpatasvir and ledipasvir, drugs used in combination with sofosbuvir to treat HCV, block the nonstructural protein NS5A involved in replication of HCV. They were also reported to inhibit 3-CL pro of SARS-CoV-2 in a docking study [87].
**Antibacterials**
  Antibacterial drugs like lymecycline, demeclocycline, doxycycline have shown to bind effectively to the binding site of 3-CL pro in computational studies conducted by Canrong Wu *et al.* [33]. Also, eravacycline, another tetracycline antibiotic used for treating intra-abdominal infections, has been identified as a possible drug candidate for SARS-CoV-2 and a potential 3-CL pro inhibitor by both virtual screening and *in vitro* analysis [101,102].
**Miscellaneous**
  Anti-hypertensive drugs like nicardipine and telmisartan and the anti-asthmatic drug montelukast have shown good binding affinity toward 3-CL pro in a virtual ligand screening where the structure of 3-CL pro was built by homology modeling [33]. Anti-tumor drugs like valrubicin and epirubicin have also been studied *in silico*, where they were shown to bind effectively to the residues in the binding pocket of 3-CL pro [92]. LinLin Zhang *et al.* recently reported the x-ray crystal structure of SARS-CoV-2 3-CL pro [86]. They have also reported the design of alpha-ketoamides exhibiting a good binding affinity to 3-CL pro [86].

##### PL-pro

**Figure 14.**
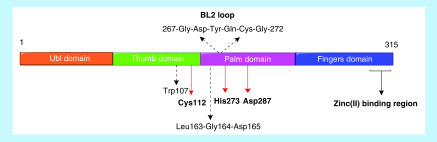
nSP3 of SARS-CoV is a multi-domain protein consisting of several domains, one of which is the PL-pro domain. It is made up of residues 315 of the nSP3 polyprotein domain. The PL-pro domain consists of an extended ubiquitin-like domain at the N terminal and a catalytic core domain. The catalytic core domain contains a thumb, palm and fingers subdomains. The active site contains a catalytic triad- Cys112-His273-Asp287 located at the interface of the thumb and palm sub-domains. Cys112 acts as nucleophile, His273 acts as a general acid-base, and Asp287 is paired with histidine helping to align it and promote deprotonation of Cys112. Trp107 aids in stabilizing the oxyanion hole. A structural zinc ion is found within a zinc-ribbon structure at the tip of the fingers region. The zinc ion is tetrahedrally coordinated by four cysteines. It is essential for catalysis because it maintains the structural integrity of PL-pro.

PL-pro processes the replicase polyproteins at three conserved cleavage sites [65]. This releases nSP1, nSP2 and nSP3, which are all essential for virus replication [33]. Additionally, PL-pro is known to reduce the host cell's immunity [33]. PL-pro functions as a protease and a deubiquitinating enzyme [103]. As depicted in the Figure 14, PL-pro of SARS-CoV is a cysteine protease with a tetrahedral zinc binding region. This region is essential for the enzyme's structural integrity and catalytic function [104]. Important sites and residues that have shown to play a role in substrate binding include-the catalytic triad Cys112, His273 and Asp287, residue Trp107, the flexible BL2 loop that interacts with the substrates and the zinc binding region. Residues instrumental in maintaining the strict substrate specificity Leu-Xaa-Gly-Gly (Xaa is Asn or Lys) in SARS-CoV PL-pro include Tyr113, Tyr274, Asn110, Leu163, Gly164, Asp165, Glu168 and Tyr265 [103,105,106].

**Figure 15.**
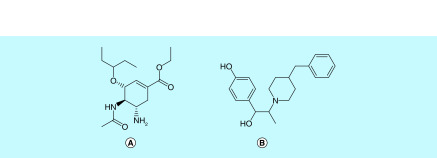
Miscellaneous therapeutics. **(A)** Oseltamivir and **(B)** ifenprodil.

Among all other targets for SARS-CoV-2, PL-pro remains the least explored. Evidently, PL-pro is an excellent target for drugs acting against SARS-CoV-2, however, no drug against PL-pro has been approved by the FDA [33]. One of the major challenges involved in designing PL-pro inhibitors is their specificity. PL-pro inhibitors could also inhibit host cell deubiquitinases, resulting in multiple side effects. Therefore, efforts have been focused on designing reversible inhibitors for PL-pro [105]. One of the key features that can be targeted is the BL2 loop. The sequence of this loop is different for different deubiquitinating enzymes and it is also involved in substrate binding [105,107]. Some antivirals that have shown a high affinity for PL-pro in computational studies include ribavirin, valganciclovir and thymidine. Notably, ribavirin showed strong hydrogen bonding and hydrophobic interaction with the enzyme. Antibacterials like chloramphenicol, cefamandole and tigecycline; muscle relaxant drugs like chlorphenesin carbamate may have a high binding affinity to PL-pro [33].

Thiopurines like 6-mercaptopurine (6-MP) and 6-thioguanine (6-TG) have proven to be effective in preclinical studies for SARS-CoV. Both 6-MP and 6-TG were found to be slow-binding, competitive, reversible inhibitors of PL-pro. The thiocarbonyl moiety was found to be crucial for inhibiting SARS-CoV PL-pro. It has been proposed that this moiety covalently binds to the catalytic cysteine residue (Cys112). Currently, both of them are used clinically for the treatment of leukemia. However, the toxicity associated with them makes them less feasible as drugs for SARS-CoV-2 [103,107]. Also, disulfiram-a drug used to treat alcohol dependence has shown good binding affinity to PL-pro and is a potential PL-pro inhibitor [37]. Disulfiram has been proposed to bind covalently to the catalytic cysteine residue in the active site. It may also bind to the zinc binding region or block the BL2 loop by binding to Cys271 in SARS-CoV PL-pro [108].

### Miscellaneous therapeutics

Oseltamivir, a neuraminidase inhibitor used to treat influenza is under Phase III clinical trials for SARS-CoV-2 (ClinicalTrials.gov: NCT04261270) [48,109].

The progression of SARS-CoV-2 is associated with a cytokine storm, hyperinflammation and ARDS. Acute lung injury and respiratory distress is one of the major reasons of mortality among SARS-CoV-2 patients [110].

The efficacy of the antibiotic-azithromycin is being studied in clinical trials for SARS-CoV-2 (ClinicalTrials.gov: NCT04332107 and NCT04359316). It has often been administered with hydroxychloroquine in clinical studies to provide a synergistic effect (ClinicalTrials.gov: NCT04332094, NCT04358081) [111]. Macrolides like azithromycin have shown modest anti-inflammatory and immunomodulatory effects in respiratory viral infections like rhinovirus (RV), RSV and influenza [112]. Thus, azithromycin may aid in controlling the cytokine storm in SARS-CoV-2 infection. Additionally, as elaborated before, it may also exhibit its activity by downregulating the expression of CD147 and blocking the invasion of SARS-CoV-2 into the host [23].

Corticosteroids are also being tested for their efficacy against some of the symptoms of SARS-CoV-2 [9,48]. Corticosteroids are used to suppress inflammation and protect lungs from injury caused by pneumonia. Acute lung injury can be caused not only by SARS-CoV-2, but also due to the immune system overdrive, and the resulting cytokine storm [113]. A preliminary clinical study on SARS patients had earlier shown beneficial effect of steroids [114]. Methylprednisone was recommended as an adjuvant therapy for severe 2019 nCoV-pneumonia patients and had showed a positive effect on the survival of SARS-CoV-2 critically ill patients in clinical trials (ClinicalTrials.gov: NCT04323592). Dexamethasone, a corticosteroid four- to five-times more potent than prednisone is in stage 4 clinical trial for treating patients with ARDS caused by SARS-CoV-2 (ClinicalTrials.gov: NCT04325061) [115].

The efficacy of corticosteroid treatment depends on three factors- the dosage, the time of application and the duration of the regimen [116]. Hence, the administration of steroids as anti-inflammatory agents in the later stage of the SARS-CoV-2 infection, when the immune system gives an excessive response, is said to be useful. But administration of corticosteroids is associated with severe side effects [117,118]. Therefore, usage of corticosteroids is still considered controversial.

Also, it has been reported that calcium signaling plays an important role in functioning of T cells and eliciting immune response of the body. Altered calcium regulation and a sustained increase in intracellular calcium concentrations can trigger cellular pathology, resulting in inflammatory syndromes [119–121]. Ifenprodil and CM4620-IE-two small molecules targeting disrupted calcium signaling are being studied for their efficacy against ARDS and lung injury caused in COVID-19 patients.

Ifenprodil is an N-methyl-D-aspartate (NDMA) receptor antagonist; a key receptor in calcium signaling and has been previously approved for treating neurological disorders [122]. CM4620-IE is a calcium release activated channel (CRAC) inhibitor. Overactivation of CRAC can cause pulmonary endothelial damage and cytokine storm in COVID-19 patients by altering cellular calcium signals. Its safety and efficacy were previously studied in regards to inflammation in acute pancreatitis [123].

Apart from small molecules, biological entities are also being administered to counter the excessive inflammatory response of the body during SARS-CoV-2 infection. Interferons are modulators of the immune system, produced in response to a viral infection [89]. Secretion of IFN stimulates the neighbouring cells to produce potent antiviral proteins [124,125]. SARS-CoV is known to be responsible for avoiding the induction of antiviral type I interferons in tissue cells [124]. This reduction in the number of interferons leads to an increased infection of viruses in the blood [124,126]. It has been reported that exogenous addition of interferons also inhibits the growth of SARS-CoV-2 [127,128]. Infection of SARS-CoV was shown to be inhibited by IFN treatment. IFN's are proposed to exhibit protective effects on the host [124]. Certain interferons, in combination with other antiviral drugs, are in various stages of clinical trials. Some of these include IFN-α2a (Pegasys^®^ and others PEGylated IFNα2a), IFN-α2b (PegIntron^®^, Sylatron^®^, IntronA^®^), IFN + Ribavirin and IFN-β1a (Avonex^®^, Plegridy^®^ [peginterferon β1a], Rebif^®^, CinnoVex^®^) [9,48].

In addition to interferons, other biological entities like monoclonal antibodies (mAbs) are also being tested in COVID-19 clinical trials to modulate the inflammatory response of the body. mAbs like tocilizumab and sarilumab function as an IL-6 receptor antagonist. Anakinra- a recombinant protein functions as an IL-1 receptor antagonist. They have been previously used for the treatment of rheumatoid arthritis and are being tested for SARS-CoV-2 (ClinicalTrials.gov: NCT04317092, NCT04335071, NCT04327388, NCT04324021) [129,130]. Siltuximab that functions as an IL-6 inhibitor is also under clinical trials for SARS-CoV-2 (ClinicalTrials.gov: NCT04329650) [131]. Leronlimab is a CC chemokine receptor (CCR5) antagonist which has shown a reduced level of IL-6 and TNF-α in patients with severe COVID-19 [132,133]. Emapalumab- a human mAb neutralizing IFN-γ has been approved for hemophagocytic lymphohistiocytosis (HLH) [134]. Its efficacy and safety in reducing hyperinflammation and respiratory distress for COVID-19 patients is currently being studied (ClinicalTrials.gov: NCT04324021). Mepolizumab is a humanized mAb targeting IL-5 and CD147 which has reportedly shown a favorable efficacy and safety profile in SARS-CoV-2 patients (ClinicalTrials.gov: NCT04275245) [53,135]. It may inhibit the entry of SARS-CoV-2 into the host by blocking CD147; a proposed alternative receptor for the spike protein [22].

Gimsilumab, lenzilumab and TJM2 are human mAbs directed against granulocyte-macrophage colony stimulating factor (GM-CSF); a pro-inflammatory cytokine and they are under COVID-19 trials (ClinicalTrials.gov: NCT04351243) [136–138]. Eculizumab, a C5 complement inhibitor and bevacizumab, an anti-VEGF drug are also under clinical trials to treat ARDS in SARS-CoV-2 patients (ClinicalTrials.gov: NCT04288713 and NCT04275414). Camrelizumab, a PD1 blocking antibody and thymosin have been reported to play a role in regulating cellular immunity during sepsis and are currently under investigation (ClinicalTrials.gov: NCT04268537) [139].

## Future Perspective

The need of the hour to mitigate the COVID-19 pandemic, is the rapid repurposing of existing drug molecules, by extensive but rapid clinical trials as is being done for remdesivir, favipiravir, chloroquine, lopinavir, ritonavir, ribavirin, oseltamivir and interferons. However, fundamental understanding of mechanisms of action of these drugs is also important, so that an understanding of combination therapy, drug synergy and toxicities are addressed at the molecular level.

In the long term, besides the development of an effective vaccine, a detailed study of already identified target receptors as well as newer ones such as the less explored peptides and other viral features such as the spike protein, the endosomal pathway, ACE2, RdRp, host cell proteases including TMPRSS2, cathepsin B, L, furin, and trypsin like proteases, viral proteases like PL-pro and 3-CL pro, is essential. Identification of druggable targets, less prone to mutations, preferably with broad spectrum activity against corona viruses, could lead to future drug molecules, with a good safety profile, for long term use.

**Executive summary**

- The SARS-CoV-2 shows a high degree of sequence conservation with SARS-CoV, which caused an outbreak in 2003 and has been extensively studied.
- The replication cycle of SARS-CoV-2 sheds light on various viral and host cell features that can be used as potential druggable targets. Repurposed drugs which were previously used to treat diseases like AIDS, Hepatitis C, influenza, asthma – among others – are strong contenders for the treatment of SARS-CoV-2. Drugs can also be designed to prevent either entry into the host cell, or replication once it enters the host cell.
- The spike (S) protein present on the viral membrane interacts with the ACE2 receptor on the host cell and is responsible for viral attachment and entry. Host cell proteases like TMPRSS2, cathepsin B,L, furin and trypsin like proteases cleave the S protein, facilitating the fusion of viral and host membranes. Thus, inhibitors of the S protein and host proteases also are potential treatment molecules for SARS-CoV-2.
- Additionally, SARS-CoV-2 can enter through endosomes, which require a low pH for functioning. Employing molecules that elevate endosomal pH, such as chloroquine and hydroxychloroquine, can inhibit this endosomal mode of entry.
- The RNA dependent RNA polymerase (RdRp) is an enzyme essential for viral replication. Molecules that are RdRp inhibitors like remdesivir, favipiravir, ribavirin which were used for the treatment of different diseases earlier, are now being repurposed for SARS-CoV-2.
- Viral proteases like the 3-CL pro and PL-pro which are responsible for the cleaving of the replicase polyproteins and are essential for viral replication can be inhibited by HIV protease inhibitors including lopinavir/ritonavir, HCV inhibitors including danoprevir as well as some antibacterials. They have shown some promise when tested against SARS-CoV-2.
- Additionally, corticosteroids, interferons and monoclonal antibodies are also under consideration for modulating immune response.
